# Nutrition knowledge, supplementation practices and access to nutrition supports of collegiate student athletes in Ireland

**DOI:** 10.1007/s00394-025-03693-y

**Published:** 2025-05-07

**Authors:** Ben Leen Smith, Conor C. Carey, Karen O’Connell, Shauna Twomey, Elaine K. McCarthy

**Affiliations:** 1 School of Food and Nutritional Sciences, University College Cork, Cork, Ireland; 2 INFANT Research Centre, University College Cork, Cork, Ireland

**Keywords:** Student athlete, Nutritional supplements, Nutrition education, Nutrition knowledge

## Abstract

**Purpose:**

To provide a comprehensive assessment of nutrition knowledge, supplementation practices and access to nutrition supports in third-level/university student athletes in Ireland.

**Methods:**

Nutrition knowledge was assessed using the Abridged Nutrition for Sport Knowledge Questionnaire (ANSKQ), with additional questions on nutrition information sources, access to nutritional supports and supplement use.

**Results:**

138 student athletes completed the online questionnaire. Participants had a mean (± SD) Total Nutrition Knowledge (TNK) score of 51.6 ± 13.7%, classed as “average”. Scores were higher in the General Nutrition Knowledge (GNK) (61.4 ± 15.4%) sub-section, while Sports Nutrition Knowledge (SNK) scores (47.2 ± 15.5%) were considered “poor”. Athletes that studied nutrition/dietetics at university level had higher GNK (69.9 ± 12.3% vs. 60.3 ± 15.5%), SNK (61.5 ± 13.7% vs. 45.3 ± 14.8%) and TNK (64.1 ± 11.8% vs. 50.0 ± 13.1%) scores than other students (all *p* < 0.05). Athletes with prior nutrition education/training also had significantly better TNK, GNK and SNK scores than those with no previous education/training (*p* < 0.001). Dietary supplements were used by 62.4% of participants, 64.0% of whom used 3 or more supplements. The most popular supplements were protein (77.9%), vitamin D (47.7%) and multivitamins (47.7%). Only 49.3% of participants had previous access to nutrition supports, but 80.4% of participants wanted more support.

**Conclusions:**

Student athletes demonstrated inadequate levels of nutrition knowledge, particularly in the areas of sports nutrition, coupled with a high prevalence of nutritional supplement use. Athletes with higher education levels and prior nutrition education/training demonstrated greater nutrition knowledge, although a high desire for more nutrition supports was reported by the athletes in this cohort.

## Introduction

Athletes have enhanced energy and nutritional requirements due to the increased physical demands of training and competition [1]. Whilst nutrition is only one of a multitude of factors affecting sports and exercise performance, optimising nutrition and dietary practices can play an important role in enhancing the nutritional status, athletic performance, recovery from exercise, general wellbeing and health of athletes [1]. Unfortunately, inadequate energy, carbohydrate, protein, and micronutrient intakes are widely reported in athletes [2–8]. Athletes have also been shown to exceed dietary recommendations for saturated fat [5], whilst poor hydration practices are commonplace [4, 9].

Multiple factors affect an athlete’s nutrition practices and dietary behaviour, including the physiological demand of exercise, appetite suppression after exercise, the feeding environment, innate food preferences, time constraints, and the cost and availability of food [10]. Nutrition knowledge is one of the few modifiable influences on dietary behaviour [11, 12]. The nutrition knowledge of athletes can be impacted by a variety of factors, including the background education of the athlete [13, 14], their previous nutrition education [3, 15, 16], access to nutrition supports [17] and sources of nutrition information [18]. Worryingly, athletes across multiple different sports and competition levels have consistently demonstrated inadequate nutrition knowledge resulting in poorer dietary practices [3, 14–16, 19, 20].

Student athletes have busy schedules due to the increased demand of balancing sport with full-time education [21]. An increased physiological, psychological and time demand may lead to the unintentional restriction of dietary intakes, with subsequent adverse effects on the athlete’s sporting and academic performance [22, 23]. Inadequate nutrition knowledge, coupled with an increased risk of food insecurity has been reported in student athletes [21], although this research is limited and has focused on US collegiate athletes predominately. Despite the amateur status of the US National Collegiate Athletics Association (NCAA), the additional supports (provision of meals, access to a dietitian etc.) available to athletes training and competing at this level may not be reflective of the wider international student athlete population [24–26].

In the US collegiate system, athletes often prioritize competition at the NCAA level and are not permitted to compete at a professional level [27]. This is in stark contrast to many athletes in Irish universities/third-level institutions that often compete at a collegiate level in addition to a normal competition schedule (local, national, regional or international level). This is particularly evident in athletes competing in Ireland’s national sports of Gaelic football and hurling/camogie where such student athletes are likely to compete for their college/university in addition to their commitments to their local club and inter-county levels [4, 14, 15, 28]. Previous data suggests that 69% of these student-county players are members of 3 or more teams, with 40% of student athletes recording less than 7 h sleep per night and 33% highlighting a lack of time as a barrier to optimal dietary intake [29, 30]. Furthermore, many athletes in these settings also compete across multiple sports. Coupled with reduced access to nutrition supports [14], student athletes in countries like Ireland are therefore likely to be at high risk for inadequate nutrition knowledge and poor dietary intakes with implications for their health and performance.

Despite this, research into this vulnerable population has been very limited, while factors influencing nutrition knowledge in this population have been largely unexplored to date. Therefore, our study aims to provide a comprehensive assessment of nutrition knowledge, supplementation practices and access to nutrition supports in third-level/university student athletes in Ireland.

## Methods

### Survey design

The nutrition knowledge, practices and access to nutrition supports of student athletes was evaluated through a web-based, self-administered survey in this cross-sectional study. The survey was composed of 59 questions, with a mixture of multiple choice and open-ended questions.

This survey used the validated abridged nutrition for sports knowledge questionnaire (ANSKQ) to assess nutrition knowledge [31, 32]. The ANSKQ consists of 35 questions in total and is composed of two sub-sections assessing general nutrition (11 questions) and sports nutrition knowledge (24 questions). The ANSKQ was composed of a mix of multiple-choice questions and questions where an athlete could “agree” or “disagree” or select “not sure”, with a score of 1 for each correct answer and a score of 0 if they selected not sure or answered incorrectly. The scoring system outlined by Trakman et al. was used to calculate scores for total nutrition knowledge (TNK), general nutrition knowledge (GNK) and sports nutrition knowledge (SNK) [31, 32]. This scoring system classified scores as: poor (0–49%), average (50–65%), good (66–75%) and excellent (76–100%) [32].

Questions around the participant’s demographic information, area of university study, level of nutrition education and use of dietary supplements were also included. Athletes provided detail on their access to and desire for nutrition supports from their sporting club/organisation. Nutrition supports were defined as “access to nutrition information only (leaflets, handouts etc)”, “access to a nutrition information session with a nutritionist/dietitian only”, “access to a 1–1 session with a nutritionist/dietitian” or “access to both a nutritionist/dietitian and a nutrition information session”. The sporting history of participants was assessed, including an evaluation of the mean hours of training per week, the receipt of a sports scholarship from the university, sports the athletes competed in and the highest level of sport that they competed in.

### Study population

Eligible participants had to be currently enrolled students in any third-level institute or university on the island of Ireland. Participants were aged 18 years or older and were active participants in any sport at any level (local, regional, university, national or international).

### Recruitment and data collection

Participants were actively recruited through promotion on social media, university-wide email lists and through contacting university sports clubs in institutions across Ireland. Participants were invited to complete the survey online through Google Forms.

### Ethics

This survey received ethical approval from the University College Cork Social Research Ethics Committee (SREC) on 6th December 2021 (Log 2021 − 199). All participants were provided with an online information sheet and provided informed consent (electronically) prior to beginning the survey. Participants were permitted to leave questions unanswered throughout the survey and retained the right to exit the survey at any point. The anonymity and confidentially of participant data was maintained throughout data collection and statistical analysis.

### Statistical analysis

Statistical analysis was carried out using SPSS for Microsoft Windows (IBM SPSS Statistics 28.0.1). Distributions of variables were tested using Kolmogorov-Smirnov tests. Descriptive analysis was carried out to generate frequency tables and calculate measures of central tendency (Mean, SD) for continuous data variables. TNK, GNK and SNK scores were calculated individually for each athlete. Pearson chi-square and Fisher’s exact tests were used for comparisons between categorical variables. For parametric data, independent sample t-tests were used to explore differences in mean TNK, SNK and GNK scores across demographic variables. Levene’s test was used to assess the homogeneity of variances during the independent sample t-test. A p value < 0.05 was considered significant.

## Results

### Sample characteristics

In total, 138 student athletes with a mean ± SD age of 22.2 ± 5.7 years completed the survey. The principal characteristics of participants are outlined in Table 1.

**Table 1:** Principal characteristics of student athletes that completed the survey (*n* = 138)

Characteristic	% Of Total Sample (Number of students)
Gender	
Male	52.9 (73)
Female	46.4 (64)
Country of Birth	
Ireland	89.9 (124)
Outside Ireland	10.1 (14)
Living Situation	
At home with parents	43.5(60)
Shared accommodation with other students	42.0 (58)
With Partner	4.4 (6)
Shared accommodation with other athletes	2.9 (4)
Shared accommodation with working professionals	2.9 (4)
With Relatives	2.2 (3)
Alone	2.2 (3)
Restrictive Diets	
Follow a restrictive diet	13.0 (18)
Do not follow a restrictive diet	87.0 (120)
Restrictive diet type	
Vegetarian / Vegan	3.6 (5)
Pescatarian	3.6 (5)
Plant Based Diet	2.9 (4)
Dairy Free	1.5 (2)
Gluten Free	1.5 (2)
Highest Level of Education	
Leaving Certificate	73.2 (101)
FETAC QQI (PLC level 5)	0.7 (1)
Advanced Certificate (NFQ level 6)	0.5 (2)
Ordinary bachelor’s degree (NFQ level 7)	1.5 (2)
Honours Bachelor’s Degree (NFQ level 8)	14.5 (20)
Master’s Degree (NFQ level 9)	8.7 (12)
Highest Competition Level	
Local/Club	37.0 (51)
University	31.9 (44)
Regional	15.9 (22)
National	6.5 (9)
International	8.7 (12)

Participants trained for an average of 8 h and 43 min (range 2–36 h) per week. Students participated in thirty-six different sporting disciplines, with 31.9% of students participating in more than one sport. The most popular sports were Gaelic football (27.5%, *n* = 38), rugby (18.1%, *n* = 25), soccer (15.2%, *n* = 21), hurling (13.8%, *n* = 19), athletics (11.6%, *n* = 16) and hockey (10.1%, *n* = 14). The highest competition level of students is outlined in Table 1. Almost half of participants competed at more than one competition level (47.1%, *n* = 65). Twenty-three students (16.7%) were in receipt of a sports scholarship.

### Nutrition knowledge

The mean Total Nutrition Knowledge (TNK) score of student athletes was 51.6 ± 13.7%, classed as “average” [31, 32]. At the individual level, 84.1% of students had TNK scores that were classed as “poor” (42.8%, *n* = 59) or “average” (41.3%, *n* = 57), with few having scores considered “good” (13.0%, *n* = 18) or “excellent” (2.9%, *n* = 4).

Students scored higher in the General Nutrition Knowledge (GNK) (61.4 ± 15.4%) sub-section, however mean scores for GNK continued to be classed as “average”. This sub-section had the largest prevalence of individual students with scores classed as “excellent” (17.4%, *n* = 24) or “good” (13.8%, *n* = 19). The mean score for the Sports Nutrition Knowledge (SNK) sub-section was considered “poor” (47.2 ± 15.5%). Most students had SNK scores that were classed as “average” (41.3%, *n* = 57) or “poor” (47.8%, *n* = 66).

Students that did not live with their parents had significantly greater GNK scores compared to students that lived with their parents (see Table 2), while living with parents had no effect on SNK or TNK scores. Competing in one sport only was associated with greater GNK scores (63.5 ± 14.9%) when compared to participants competing in multiple (≥ 2) sports (56.8 ± 15.5%, *p* = 0.016), although no differences were seen in SNK or TNK scores. GNK, TNK or SNK scores did not differ significantly depending on the type of sport that participants were competing in (i.e. GAA, rugby, soccer, athletics or hockey). There was also no significant difference in nutrition knowledge between athletes competing in individual sports (i.e., athletics, weightlifting etc.) versus those in team sports (rugby, GAA, soccer etc.).

**Table 2:** Mean ± SD nutrition knowledge scores for total sample and across demographic and sporting characteristics

	GNK Score (%)	*p*-value	SNK Score (%)	*p*-value	TNK Score (%)	*p*-value
Total Sample	61.4 **±** 15.4		47.2 **±** 15.5		51.6 **±** 13.7	
Gender						
Male (*n* = 73)	60.2 ± 16.9	0.348	47.7 ± 16.6	0.661	51.6 ± 15.1	0.992
Female (*n* = 64)	62.6 ± 13.6		46.5 **±** 14.4		51.6 ± 12.1	
Living Situation						
Living with parents (*n* = 60)	57.9 ± 14.8	0.018*	45.6 ± 15.0	0.288	49.4 ± 13.0	0.097
Not living with parents (*n* = 78)	64.1 ± 15.4		48.4 ± 15.9		53.3 ± 14.1	
Restrictive Diet						
Followed a restrictive diet (*n* = 18)	65.2 ± 13.3	0.269	50.9 ± 18.2	0.271	55.4 ± 14.5	0.213
Did not follow a restrictive diet (*n* = 120)	60.8 ± 15.7		46.6 ± 15.1		51.1 ± 13.6	
Multiple Sports						
One sport only (*n* = 94)	63.5 ± 15.5	0.016*	47.8 ± 12.4	0.493	52.7 ± 11.4	0.169
More than one sport (*n* = 44)	56.8 ± 15.0		45.8 ± 16.8		49.3 ± 14.6	
Sports Scholarship						
Yes (*n* = 23)	56.5 ± 17.5	0.097	45.1 ± 12.7	0.489	48.7 ± 16.0	0.262
No (*n* = 115)	62.4 ± 14.8		47.6 ± 16.0		52.2 ± 13.8	
Competition Level						
Regional, National, or International (*n* = 43)	57.5 ± 16.1	0.046*	48.1 ± 16.4	0.648	51.0 ± 15.1	0.728
Local or University (*n* = 95)	63.2 ± 14.9		46.8 ± 15.2		51.9 ± 13.1	

*Significantly different scores between groups as determined by an independent-samples t-test (*p* < 0.05)

### Educational background

Participants were currently enrolled or had completed degrees in 14 different subject areas, including Business (15.2%, *n* = 21), Science (13.7%, *n* = 19) and Medicine/Health (13.0%, *n* = 18). Only 11.6% of participants were enrolled in a nutrition or dietetics degree programme. Athletes that had already completed an honours bachelor’s degree or master’s degree prior to taking the survey had significantly greater GNK (69.0 ± 16.6% vs. 59.1 ± 14.3%) and TNK (56.4 ± 16.4% vs. 50.2 ± 12.5%) scores (all *p* < 0.05) in comparison to those whose highest level of education at the time of the survey was the Leaving Certificate.

Students enrolled in a nutrition or dietetics programme at university level had mean GNK (69.9 ± 12.3%) and TNK (64.1 ± 11.8%) scores that were classed as “good” and SNK scores (61.5 ± 13.7) classed as “average” (Table 3). The GNK (60.3 ± 15.5%) and TNK (50.0 ± 13.1%) scores of students enrolled in programmes outside of nutrition/dietetics were considered “average”, whilst SNK scores (45.3 ± 14.8%) were classed as “poor”. The mean GNK, SNK and TNK scores of nutrition/dietetics students were significantly higher than the mean scores of students enrolled in non-nutrition or dietetic related degree programmes (all *p* < 0.05) (Table 3).

**Table 3:** Mean ± SD nutrition knowledge scores by educational background

	GNK Score (%)	*p*-value	SNK Score (%)	*p*-value	TNK Score (%)	*p*-value
Nutrition/Dietetics Degree Enrolment						
Yes (*n* = 16)	69.9 ± 12.3	0.019*	61.5 ± 13.7	< 0.001^*^	64.1 ± 11.8	< 0.001^*^
No (*n* = 122)	60.3 ± 15.5		45.3 ± 14.8		50.0 ± 13.1	
Science, Medicine or Nutrition/Dietetics Degree Enrolment						
Yes (*n* = 53)	67.2 ± 15.0	< 0.001*	52.8 ± 14.1	< 0.001*	57.4 ± 12.6	< 0.001*
No (*n* = 85)	57.8 ± 14.6		43.6 ± 15.4		48.1 ± 13.2	
Medicine Degree Enrolment						
Yes (*n* = 18)	70.2 ± 14.2	0.009*	51.4 ± 13.9	0.216	57.3 ± 13.2	0.060
No (*n* = 120)	60.1 ± 15.2		46.5 ± 15.7		50.8 ± 13.6	
Highest Education Level Attained						
Level 8 or Higher (*n* = 32)	69.0 ± 16.6	< 0.001*	50.7 ± 18.5	0.147	56.4 ± 16.4	0.024*
Level 7 or Lower (*n* = 106)	59.1 ± 14.3		46.1 ± 14.4		50.2 ± 12.5	

*Significantly different scores between groups as determined by an independent-samples t-test (*p* < 0.05)

### Nutrition education or training

Over half (50.7%, *n* = 70) of the participants had prior exposure to some form of nutrition education or training (i.e., university course, university module, short/online nutrition course, secondary school education etc.). Participants who had previously received any nutrition education or training had significantly higher mean GNK (67.2 ± 12.2% vs. 56.0 ± 16.2%), SNK (51.7 ± 13.6% vs. 42.9 ± 16.1%) and TNK (56.6 ± 11.5% vs. 47.0 ± 14.1%) scores than those that had not received any nutrition education or training (*p* < 0.001). Differences in GNK (*p* < 0.001), TNK (*p* = 0.002) and SNK (*p* = 0.035) remained when excluding students enrolled in a third-level nutrition or dietetics programme.

### Sources of nutrition information

The most common sources of nutrition information reported by participants included the internet (63.8%, *n* = 89), social media (45.7%, *n* = 63), coach/personal trainer (36.2%, *n* = 43) and teammates/other athletes (35.5%, *n* = 50). Books/scientific literature were listed as a source of nutrition information by 33.3% of students. The use of books/scientific literature as a source of nutrition information was associated with significantly greater GNK (71.2 ± 13.7% vs. 56.5 ± 13.9%), SNK (53.5 ± 14.1% vs. 44.0 ± 15.3%) and TNK (59.1 ± 11.7% vs. 47.9 ± 13.2%) scores in comparison to those not using this source of information (all *p* < 0.001).

Thirty-four students (24.6%) had previously consulted a qualified nutritionist or dietitian as a source of nutrition information. These participants recorded greater GNK (67.2 ± 13.8% vs. 58.9 ± 15.5%), SNK (53.4 ± 13.9% vs. 44.5 ± 15.5%) and TNK scores (57.7 ± 12.2% vs. 49.1 ± 13.6%) than students that did not use a nutritionist/dietitian to source their information (all *p* < 0.001). There was no difference noted by using the other sources of nutrition information on GNK, SNK or TNK scores.

### Access to nutrition supports

Sixty-eight students (49.3%) had received specific nutrition support from their sports club/organisation in the past. Of the students that had received nutrition support; 20.6% had access to a nutritionist or dietitian on a one-to-one basis. Athletes in receipt of a sports scholarship had significantly greater access to nutrition supports from their club/organisation (78.3% vs. 43.5%, *p* = 0.002) and had greater access to a nutritionist/dietitian on a one-to-one basis (47.8% vs. 15.7%, *p* < 0.001).

There was a high demand for more nutrition support from athletes, with 80.4% (*n* = 111) of the sample stating that they would like more nutrition support from their sporting club/organisation. A total of 101 of these athletes (91%) specified that they would want access to a qualified nutritionist or dietitian, whilst 66 athletes (47.8%) highlighted a demand for more nutrition information from the club via leaflets and resources. Participants cited a need for improved education or guidance on a variety of topics, including “*Healthy Recipes/Meal Ideas”* (56.5%, n = 78), *“Protein Powders/Supplements”* (48.6%, n = 67), *“Carbohydrate Loading”* (47.1%, n = 65), *“Weight Gain/Weight Loss”* (40.6%, n = 56) and *“Creatine”* (26.8%, n = 48).

Access to an information session with a qualified nutritionist or dietitian alone was associated with increased SNK scores, but not with improvements in GNK or TNK scores (Table 4). Access to any other nutrition supports was not associated with differences in nutrition knowledge scores, as outlined in Table 4.

**Table 4:** Mean ± SD nutrition knowledge scores by access to nutrition supports

	GNK Score (%)	*p*-value	SNK Score (%)	*p*-value	TNK Score (%)	*p*-value
Received Nutrition Support from sports club/organization						
Yes (*n* = 68)	61.2 ± 14.5	0.901	49.3 ± 12.8	0.116	53.0 ± 11.8	0.242
No (*n* = 70)	61.6 ± 16.3		45.1 ± 17.6		50.1 ± 15.3	
Access to nutrition information (leaflets etc.) *only*						
Yes (*n* = 18)	62.1 ± 18.0	0.831	45.1 ± 15.4	0.555	50.5 ± 14.0	0.702
No (*n* = 120)	61.3 ± 15.1		47.5 ± 15.6		51.8 ± 13.7	
Access to a qualified nutritionist/dietitian (one to one basis) *only*						
Yes (*n* = 29)	62.1 ± 14.4	0.793	49.6 ± 13.1	0.349	53.5 ± 12.5	0.413
No (*n* = 109)	61.2 ± 15.7		46.5 ± 16.1		51.1 ± 14.0	
Access to information session with a qualified nutritionist or dietitian *only*						
Yes (*n* = 21)	59.3 ± 11.7	0.410	52.4 ± 9.4	0.019^*^	54.6 ± 8.3	0.133
No (*n* = 117)	61.8 ± 16.0		46.2 ± 16.2		51.1 ± 14.4	
Access to information session *and* one to one session with a qualified nutritionist/dietitian						
Yes (*n* = 15)	63.0 ± 14.0	0.665	51.4 ± 14.6	0.265	55.1 ± 13.3	0.309
No (*n* = 123)	61.2 ± 15.6		46.7 ± 15.6		51.2 ± 13.8	

*Significantly different scores between groups as determined by an independent-samples t-test (*p* < 0.05)

### Nutritional supplement use

**Fig. 1:**
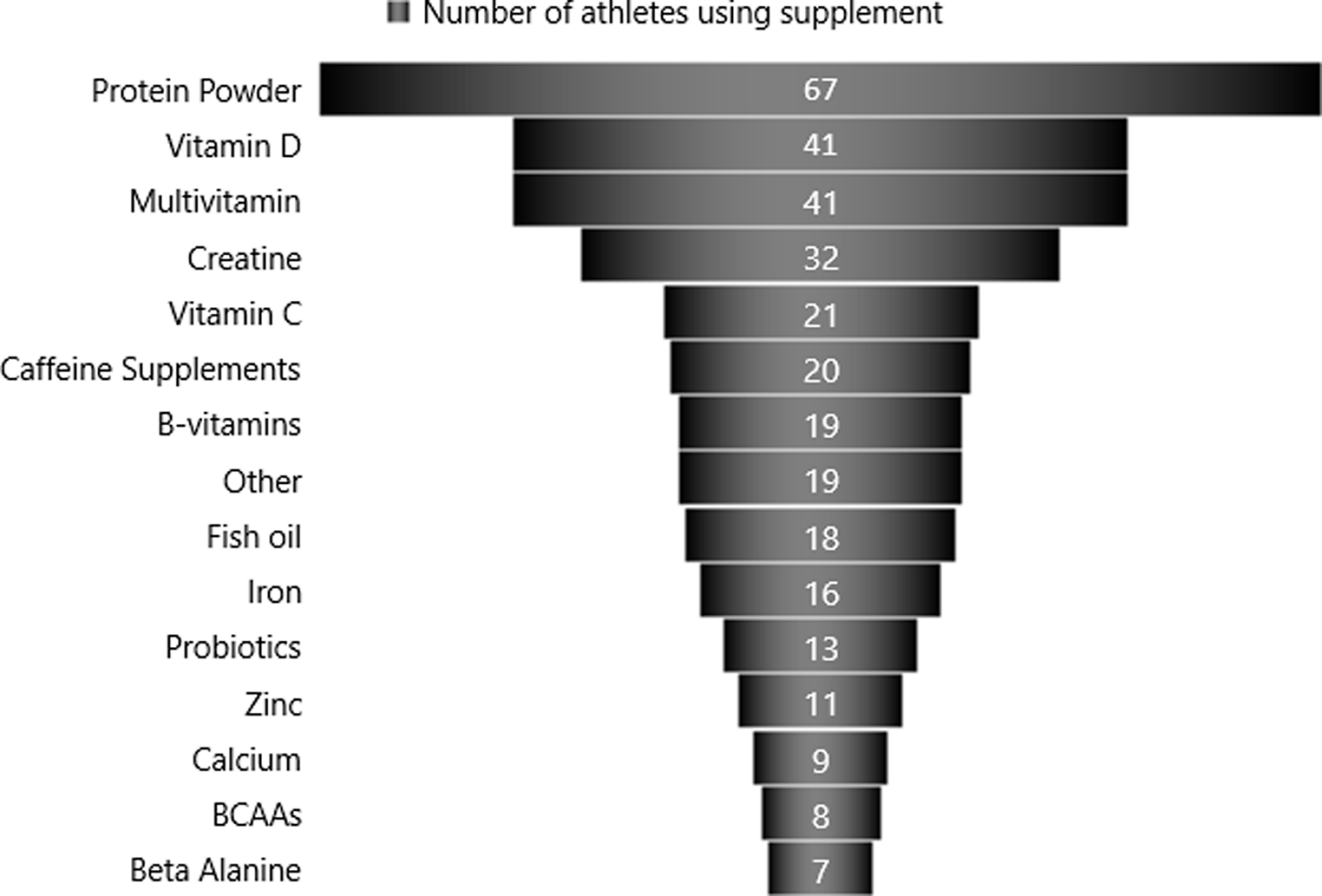
An overview of the dietary supplements utilized by student athletes in this study

**Fig. 2:**
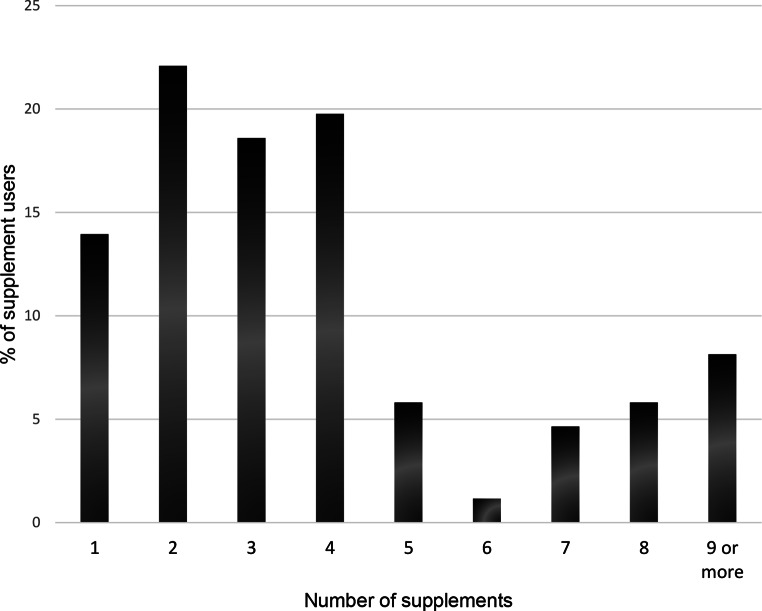
Quantity of different dietary supplements used by student athlete supplement users

Supplements were used by eighty-six students (62.3%), with twenty-four different types of supplements reported within the survey sample. Of the athletes that used supplements, as outlined in Fig. 1, the most popular supplement was protein powders/supplement used by 77.9% (*n* = 67), followed by vitamin D (47.7%, *n* = 41), multivitamins (47.7%, *n* = 41), creatine (37.2%, *n* = 32), vitamin C (24.4%, *n* = 21) and caffeine supplements (23.3%, *n* = 20). In total, 64.0% (*n* = 88) of supplement users reported using 3 or more supplements and 8.1% (*n* = 11) reported using 9 or more supplements (Fig. 2).

Of the students that used supplements, the most common reasons for supplementation were “*To avoid nutrient deficiency*” (66.3%, *n* = 57), “*To improve sports performance”* (62.8%, *n* = 54), “*To enhance recovery after sports performance”* (61.6%, *n* = 53) and “*To meet increased nutrient requirements”* (37.2%, n = 32).

Athletes that used supplements had significantly higher GNK, SNK and TNK scores than those that did not use supplements (Table 5). A similar association was reported for those that used protein supplements, but no differences in knowledge levels were observed in creatine or iron supplement users. Mean TNK scores for supplement users were considered “average”, whereas the mean TNK scores of students who did not use supplements were classed as “poor”.

**Table 5:** Mean ± SD nutrition knowledge scores by nutritional supplement use

	GNK Score (%)	*p*-value	SNK Score (%)	*p*-value	TNK Score (%)	*p*-value
Any Nutritional Supplement Use						
Yes (*n* = 86)	63.6 ± 15.3	0.028*	51.1 ± 13.6	< 0.001*	55.1 ± 12.4	< 0.001*
No (*n* = 52)	57.7 ± 14.9		40.6 ± 16.3		46.0 ± 14.0	
Protein Supplement Use						
Yes (*n* = 67)	64.3 ± 15.8	0.030*	52.6 ± 13.9	< 0.001*	56.3 ± 13.1	< 0.001*
No (*n* = 71)	58.6 ± 14.6		42.1 ± 15.4		47.3 ± 12.9	
Creatine Supplement Use						
Yes (*n* = 32)	63.6 ± 18.5	0.350	50.7 ± 12.8	0.147	54.7 ± 13.0	0.146
No (*n* = 106)	60.7 ± 14.4		46.1 ± 16.2		50.7 ± 13.8	
Iron Supplement Use						
Yes (*n* = 16)	56.3 ± 16.3	0.156	51.3 ± 11.5	0.258	52.9 ± 11.7	0.706
No (*n* = 122)	62.1 ± 15.2		46.6 ± 15.9		51.5 ± 14.0	
Multivitamin Use						
Yes (*n* = 41)	63.4 ± 16.3	0.319	52.9 ± 14.4	0.005*	56.2 ± 13.3	0.011*
No (*n* = 97)	60.5 ± 15.0		44.8 ± 15.4		49.7 ± 13.5	

*Significantly different scores between groups as determined by an independent-samples t-test (*p* < 0.05)

Athletes that used protein supplements recorded significantly higher scores (56.7 ± 18.5%) in the 10 questions relating to protein on the ANSKQ than those that did not use protein supplements (45.2 ± 17.3%, *p* < 0.001). Athletes using multivitamins had significantly greater SNK and TNK scores compared to those that did not use a multivitamin, with SNK (44.8 ± 15.4%) and TNK (49.7 ± 13.5%) scores categorised as “poor” in those not using multivitamins.

## Discussion

In one of the first investigations into this vulnerable population, collegiate student athletes in Ireland displayed inadequate levels of nutrition knowledge, which was underpinned by poor sports nutrition knowledge in particular. Athletes with higher education levels and prior nutrition education/training demonstrated better nutrition knowledge. Supplement use was high amongst student athletes in this population, with many athletes utilising multiple supplements. Despite a high demand for further nutrition support, many student athletes in Ireland appeared to have limited access to meaningful nutrition supports.

Despite displaying “average” nutrition knowledge, student athletes in our study had better knowledge levels using the ANSKQ assessment tool than non-elite Australian football and netball players (47.0 ± 12.0%) [32], Gaelic football players (46.0 ± 11.8% and 47.8 ± 11.0%) [14, 15], Jordanian athletes (35.1 ± 12.7%) [20] and recreational athletes in Brazil (50.7 ± 16.2%) [19]. In contrast, our athletic population had lower knowledge levels than previous investigations using alternative assessment tools to the ANSKQ in NCAA Division III footballers (55.2 ± 16.3%) [3], Finnish endurance athletes (72.7 ± 8.8%) and cross-country skiers (76.0 ± 7.3%) [33, 34] and elite soccer players in Australia (56.0 ± 8.5%) [35]. Nutrition knowledge scores in our population were slightly lower than alternative assessments among university students in Malaysia and the US, but higher than student athletes in Iran [36–38]. The use of multiple different nutrition knowledge assessment tools across different studies is a major limitation of this field and does not allow for a direct comparison of results with our sample.

Athletes recorded poor sports nutrition knowledge scores, consistent with previous findings in Australian football and netball players (35.0% ± 18.0%) [32], Gaelic football players in Ireland (40.4 ± 13.0% and 44.6 ± 12.3%) [14, 15] and recreational athletes in Brazil (45.2 ± 18.6%) [19] which also used the ANSKQ. Previous studies of elite and non-elite soccer and Australian football players reported better sports nutrition knowledge than general nutrition knowledge, but these studies used alternative nutrition knowledge assessment tools [35, 39]. Athletes in our study scored poorly on questions relating to carbohydrate and protein requirements after exercise, carbohydrate sources and the role of certain micronutrients. These findings are comparable to other populations, where knowledge on carbohydrate and protein requirements, micronutrients and supplements remain inadequate [4, 40–43]. This is concerning, as inadequate sports nutrition knowledge appears to translate into poorer dietary practices in athletes, particularly evidenced by inadequate carbohydrate intakes to fuel exercise [2–4, 28, 39, 44].

As has been demonstrated in previous investigations in both athletes and non-athletes [13, 14], higher background education levels resulted in greater nutrition knowledge in this cohort. Higher levels of general education may act as a reflection of increased critical thinking skills and the ability to differentiate between both reliable and untrustworthy nutrition information, particularly given the reliance upon the internet and social media as sources of nutrition information [45]. Unsurprisingly, athletes studying the disciplines of medicine or nutrition/dietetics had better nutrition knowledge than those studying other disciplines. This has been reported elsewhere, including amongst medical students in Iran and Portugal [46, 47] and nutrition and dietetics students in Spain [48].

Prior exposure to nutrition education or training (for example a university module or secondary school subject such as biology/home economics) was associated with better nutrition knowledge in our cohort of student athletes, consistent with findings from two studies conducted in Gaelic footballers in Ireland previously [14, 15]. This finding complements previous reports of successful nutrition education interventions conducted amongst student athletes in the US [7, 49, 50] and further highlights the potential benefit of a structured nutrition education programme for third-level student athletes. The reliance on social media and the internet as key sources of nutrition information [51], coupled with the promise of online nutrition education interventions [11] in other cohorts may highlight a vehicle for future interventions.

Poor sports nutrition knowledge scores as reported in this study and others [14, 15, 20, 32] may be reflective of a larger issue surrounding the communication of the sports nutrition literature to all athletes. The sources of nutrition information used by athletes has previously been shown to influence levels of nutrition knowledge [18], with many studies in addition to ours highlighting the importance of the internet, social media and coaches/personal trainers as sources of information [3, 40, 52, 53]. Some studies have found that coaches demonstrated inadequate nutrition knowledge [54, 55], while others suggest that sports practitioners have adequate nutrition knowledge [20, 40]. While no effect of coaches/trainers on nutrition knowledge was observed in this study, the use of a nutritionist or dietitian as a source of nutrition information did result in greater nutrition knowledge amongst our student athletes. Interestingly, the use of books or scientific literature was identified as a source of nutrition information by over a third of our student athlete population. Student athletes may have greater exposure to such sources as part of their education programmes than other athlete groups, as has previously been observed in student athletes in Jordan and Malaysia [20, 36]. These findings highlight the potential of high-quality evidence-based nutrition information to improve nutrition knowledge, however efforts should be made to increase the accessibility of these resources to all athletes.

Levels of access to nutrition supports were inadequate in this study, with less than half of student athletes reporting having had access to nutrition supports from their sports club/organisation. Once-off nutrition supports were relatively unsuccessful, but access to an information session with a qualified nutrition professional was associated with better sports nutrition knowledge in our cohort. Individual consultations with a nutritionist or dietitian alone were shown to increase nutrition knowledge scores, carbohydrate, and protein intakes amongst university athletes in previous investigations [7, 56], although this alone was not associated with greater total nutrition knowledge in our study or by Trakman and colleagues in Australian footballers [13]. Trakman and colleagues have previously suggested that this may be due to an athletes over reliance on their nutritionist or dietitian when making food choices [13]. Therefore, interventions likely need to focus on a mix between individualised nutrition counselling and the delivery of lectures/information sessions to athletes for the most benefit. Such approaches have previously demonstrated efficacy in improving nutrition knowledge and dietary behaviour [11, 57].

Nutrition and its potential role in improving athletic performance is of clear value to this population, as there was a very high demand for further nutrition support reported in our study. In the USA, the NCAA reports that its colleges spend a total of 3.5 billion dollars per year on division 1 student athletes, including access to registered dietitians, provision of unlimited meals, access to additional tutors, academic advisors and sport psychologists [26]. Despite this high level of support, 71% of male and 88% of female NCAA athletes reported feeling mentally exhausted [58] while 61% experience daytime fatigue 3 or more days per week [26]. Whilst similar impact assessments are limited for Irish student athletes, this level of support is unfortunately not reflective of most of the athletes in our population. The disparity in supports provided to Irish student athletes is highlighted by data suggesting that 40% of inter-county (highest level of competition) Gaelic footballers had to sit repeat exams and 14% had to repeat a full year of university, compared to 6% of the wider student population [29]. Despite competing and training at an “elite” level, the lack of consistent access to elite level nutrition supports may prove troublesome, with inadequate energy, carbohydrate and micronutrient intakes previously reported in these athletes [4, 28]. Our data suggests that this issue is perhaps exacerbated even further in these student athletes. Further considerations are also needed for athletes who compete in multiple sports, with almost a third of our population competing across multiple sports.

Almost two-thirds of athletes in our study reported nutritional supplement use, which is lower than prevalence data from US and Canadian collegiate athletes [24, 59] but similar to rates reported in a broader Irish athletic population and a meta-analysis of mixed athletic groups [60, 61]. These rates of supplement use are much higher than the levels reported in the general adult population of Ireland [62], perhaps highlighting the apparent importance given to supplements by athletes for achieving peak sports performance. Supplement users in our study had significantly higher total, general and sports nutrition knowledge scores in comparison to non-supplement users. It is possible that these effects could be bi-directional in nature, as better nutrition knowledge may have resulted in an increased frequency and effective use of supplements. While a food first approach to sports nutrition is widely advocated for, particularly for optimising muscle protein synthesis, protein supplements were the most consumed supplement in our student athlete population [1, 63–66]. The value of protein supplements to this population is mirrored by secondary school and collegiate athletes in the US and Ireland [53, 67, 68], perhaps acting as a reflection of the time constraints facing student athletes and the perceived convenience of these supplements in contrast to preparing high-protein snacks and meals at home. Importantly, despite supplement users displaying increased nutrition knowledge, over a third of our supplement users were unaware that all supplements are not batch tested to identify cross contamination with prohibited substances. This is alarming given the current landscape around doping but also given that over two-thirds of supplement users in our study were taking 3 or more supplements.

The representation of athletes across a multitude of different sports was a strength of this study, with athletes competing at a variety of different competition levels which provides a much-needed wider representation of the student athlete population. The use of the ANSKQ, a validated tool for the assessment of sports nutrition knowledge is another strength of our study. However, a major limitation of this field is the use of multiple different nutrition knowledge assessment tools which doesn’t easily allow for comparisons to be made between studies. The delivery of the nutrition knowledge assessment online was a limitation of this study, as we were unable to ensure that athletes had not consulted with external resources when completing the assessment, but this was necessitated due to COVID-19 restrictions at the time of survey delivery. This may have led to higher scores using the ANSKQ in our study population, however, online delivery of such assessments including the ANSKQ is typical for other studies also. Participants were predominantly recruited through one university and were mostly born in Ireland. This study did not collect dietary intake data from participants, limiting explorations between knowledge levels and actual dietary practices.

## Conclusion

Student athletes are vulnerable to nutritional issues, impacting their health and athletic performance but clear gaps exist in the nutrition knowledge of this cohort, particularly in sports nutrition knowledge. Access to nutrition support was inadequate in our cohort, but athletes with prior nutrition education/training exposure had better nutrition knowledge than those with no prior education. Supplement use was high within our cohort and may act as a reflection of nutrition attitudes within this group, highlighting a need to further explore the motivators for athlete supplement use.

This study highlights the need for high-quality nutrition education supports to address gaps in nutrition knowledge within this group. The reliance upon the internet and social media as sources of nutrition education may highlight a vehicle for such nutrition education interventions. The challenges with delivering such approaches on a wider scale are noteworthy but given both the desire and need for such support from athletes, efforts to address these challenges need to be prioritised.
